# A joint modelling approach for multistate processes subject to resolution and under intermittent observations

**DOI:** 10.1002/sim.7149

**Published:** 2016-10-17

**Authors:** Sean Yiu, Brian Tom

**Affiliations:** ^1^ MRC Biostatistics Unit Cambridge CB2 0SR U.K.

**Keywords:** intermittent observations, joint modelling, multistate model, psoriatic arthritis, resolution

## Abstract

Multistate processes provide a convenient framework when interest lies in characterising the transition intensities between a set of defined states. If, however, there is an unobserved event of interest (not known if and when the event occurs), which when it occurs stops future transitions in the multistate process from occurring, then drawing inference from the joint multistate and event process can be problematic. In health studies, a particular example of this could be resolution, where a resolved patient can no longer experience any further symptoms, and this is explored here for illustration. A multistate model that includes the state space of the original multistate process but partitions the state representing absent symptoms into a latent absorbing resolved state and a temporary transient state of absent symptoms is proposed. The expanded state space explicitly distinguishes between resolved and temporary spells of absent symptoms through disjoint states and allows the uncertainty of not knowing if resolution has occurred to be easily captured when constructing the likelihood; observations of absent symptoms can be considered to be temporary or having resulted from resolution. The proposed methodology is illustrated on a psoriatic arthritis data set where the outcome of interest is a set of intermittently observed disability scores. Estimated probabilities of resolving are also obtained from the model. © 2016 The Authors. *Statistics in Medicine* Published by John Wiley & Sons Ltd.

## 1 Introduction

In many pathological processes, patients may completely recover and therefore be free of any further symptoms. This, for example, could be due to an appropriate dose or medication being found, a disease process burning out 1, 2 or even because of a change of environments 3.

For a set of intermittently observed psoriatic arthritis (PsA) patients, the primary aims of this paper are to characterise the rates of progression between a set of disability states and to consider the extent to which resolution occurs. Some complications in this scenario arise from the unknown times in which the disability status changes (transitions are interval censored) and also not knowing if and when a patient has resolved. Note that the latter complication is in contrast to the standard time‐to‐event setting because it is generally known if the event of interest has occurred, such as death, even if the occurrence time is not known. For this situation, we propose an expanded multistate model, which can naturally handle intermittent observations, that composes of (i) a latent absorbing state representing resolution, (ii) a partially latent transient state of temporary non‐disability (therefore together representing no disability as a disjoint union of temporary and permanent non‐disability/resolution) and (iii) various transient states of disability severity. The temporary non‐disability state is partially latent because observations of no disability is known to be temporary if observations of disability have been observed afterwards. Implicitly, because non‐disability is partitioned into a latent and partially latent state representing resolved and temporary non‐disability, respectively, inference on each event can be obtained explicitly, and the uncertainty of not knowing if a patient has resolved can easily be accounted for when constructing the likelihood; observation of no disability can be considered as either temporary or permanent.

In survival analysis, mixture models have been frequently used in studies where a proportion of long‐term survivors are believed to have been cured at a single point in time, such as, straight after surgery, for example 4, 5, 6. These models estimate the latent probability of being cured for each patient and the survival distribution for the uncured patients. Yamaguchi 7 examined a further interesting notion by characterising the latent failure time at which a patient becomes cured, and this was motivated by the possibility of patients becoming cured at different points in time. This was performed using a three‐state competing risk model where the states were alive and not cured (transient state), cured, and death (both absorbing states), thus the alive state is dicho tomised into the disjoint union of a partially latent alive and not cured state and a latent cured state. Observations of alive were then considered to be in either the cured state or alive and not cured state when constructing the likelihood, therefore accounting for the uncertainty of not knowing if a patient has been cured. Yamaguchi 8 also explored last episode data where the last occurrence of a repeatable event is of interest. As this event time (the time of the last episode), which is analogous to the failure time into the resolved state, is unknown, a time‐varying latent indicator variable was incorporated into the intensity, and this represented the uncertainty about the last observed occurrence being also the last possible event. Shen and Cook 3 studied recurrent event processes that are subject to resolution. Their approach was similarly based on time‐varying latent indicator variables, and they suggested the use of an Expectation‐maximisation (EM) algorithm to facilitate the model fitting procedure. All of these analyses were based on knowing the times (subject to right censoring in the survival cases) when the events, apart from resolution, occurred.

Cook *et al.* 9 considered a generalised mover–stayer model for intermittently observed multistate processes. The model (based on time‐invariant latent indicator variables) consisted of a mixture of nested continuous‐time multistate processes in which sub‐models were defined by constraining some transitions to zero between two or more states of a full model. This methodology would then allow for the possibility that some states were permanently transient (i.e. positive intensity) for some patients and the same states were permanently absorbing (i.e. zero intensity) for others. However, all states were assumed non‐latent and therefore observable, even though it may not be known which intensities are zero. If this methodology was applied to our illustrative example, the observable non‐disability state could only either always represent temporary non‐disability (i.e. intensity out of the observable non‐disability state is positive) or resolved (i.e. intensity out of the observable non‐disability state is zero) for each patient. As such, patients who have ever been observed to transition from the non‐disability state to a disability state will always be represented as having temporary non‐disability when in the non‐disability state even if they have resolved; the intensity from the non‐disability state must be permanently positive to reflect the previous transition(s) out of the non‐disability state. Thus, characterising the extent to which these patients resolve (those who have previously been observed to transition out of the non‐disability state), in which there are many in the illustrative example, is not facilitated by this methodology. Instead, if the observable non‐disability state is partitioned into a latent absorbing resolved state and partially latent transient temporary non‐disability state, patients could be characterised as resolved (by the resolved state) at any point in time regardless of their observed history. We also note that Shen and Cook 10 recently extended their model in 3 for recurrent event processes to allow for an intermittent observation scheme.

The remainder of this paper is organised as follows. In the next section, disability in psoriatic arthritis is briefly introduced. In Section 2, the notation and methodology is described. Section 3 provides a short simulation study to facilitate discussions on the expected discrepancies (with regard to parameter estimates and confidence intervals) that occur when observations of no disability are known and unknown to correspond to resolved. Section 4 applies the methodology, and concluding remarks are made in Section 5.

### 1.1 Disability in psoriatic arthritis

Psoriatic arthritis patients generally experience both skin and joints manifestations of the disease. For some patients, this can lead to severe physical functional disability. In PsA, physical disability is predominantly measured using the Health Assessment Questionnaire (HAQ), which yields a score between 0 (no disability) and 3 (completely disabled), with increments of 0.125. At the University of Toronto PsA Clinic 11, 597 patients have completed the HAQ on at least two occasions, with the average number of responses being 6.41 (ranging from 2 to 18). The mean and median intervisit times of these patients were 1 year and 6 months and 1 year and 1 month, respectively, whilst their mean follow‐up time was 7 years and 6 months with an interquartile range (IQR) of 9 years and 2 months. In total, there were 3829 observations with the average HAQ score being 0.61 (standard deviation (SD) of 0.68).

**Figure 1:**
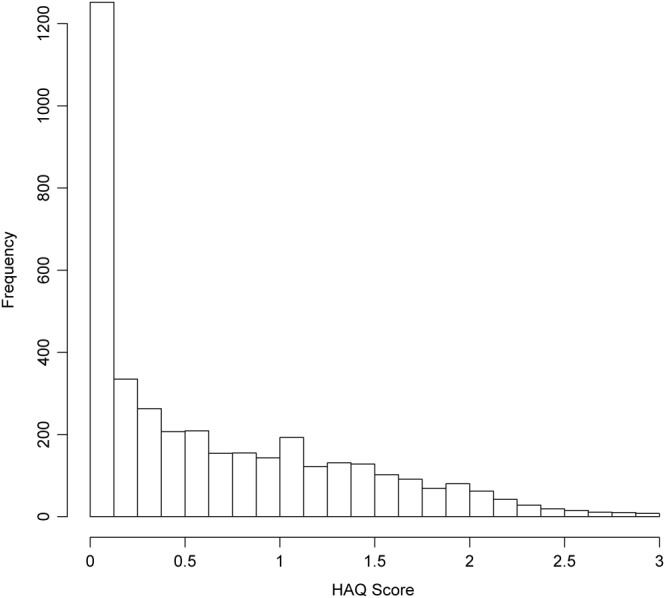
A histogram describing the frequencies of the Health Assessment Questionnaire (HAQ) scores in our data.

**Figure 2:**
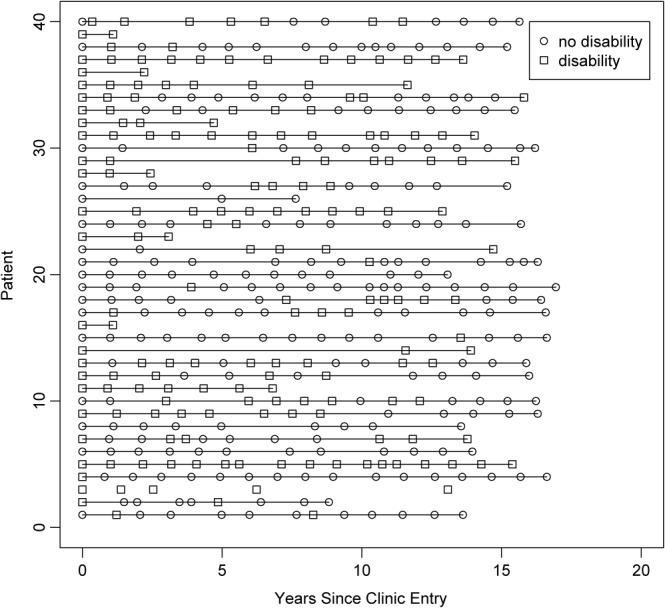
A plot of the disability trajectories for 40 patients. The circles and squares represent observations of zero and non‐zero Health Assessment Questionnaire scores, respectively.

A notable feature of this data set is the large number of zero observations (32*%*, 1252 of 3829 observations), as seen in Figure 1, which displays the frequencies of HAQ scores. On further examination, it can be seen that 708 (57*%*) of these zeros lie consecutively between the last time some patients were observed to be in a state of disability and the end of their follow‐up period. This occurred for 179 patients (30*%*, 179 of 597 patients) where the mean time since their last observed disability state was 4 years and 7 months with an IQR of 4 years and 9 months. For a subset of the data, this finding is illustrated in Figure 2. It is a general belief that some patients will gradually adapt to their disability status, and as a consequence will not report any further disability after a period of adjustment. For this reason, and others, persuasive arguments can be made about the clinical plausibility that certain patients become resolved. These clinical and empirical considerations, in part, motivated the development of the methodology to follow.

## 2 Methodology

**Figure 3:**
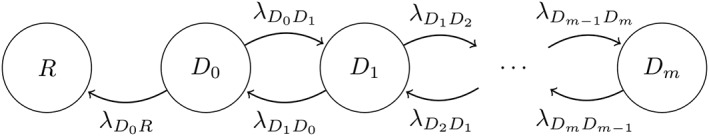
Multistate diagram that describes the disability and resolution processes jointly.

This paper focuses on the situation where there are *n* patients whose disability process is subject to resolution. Denote the disability process at time *t*, *X*(*t*), which can take a set of states {*R*,*D*_0_,*D*_1_,…,*D*_*m*_}. The multistate model with state space displayed in Figure 3 can then be used to describe the progression of disability through these states. Here, state *R* represents a resolved state where upon entry, no further disability can be gained. Hence, it is appropriately characterised as an absorbing state. Next, state *D*_0_ denotes a temporary non‐disability state as further transitions into disability are possible. Finally, states {*D*_1_,…,*D*_*m*_} represent increasing levels of disability severity where each state is only accessible via adjacent states. This assumption in continuous time is not restrictive as transitions between non‐adjacent states over two successive visits in the data would necessarily have been via the unobserved adjacent states in the period between these visits.

Suppose for patient *i* realisations of the disability states ${\left\{s_{ij}\right\}}_{j=0}^{m_{i}}$ and a vector of covariates ${\left\{z_{ij}\right\}}_{j=0}^{m_{i}}$ are recorded at clinic visits occurring at times ${\left\{t_{ij}\right\}}_{j=0}^{m_{i}}$. Covariate effects can be incorporated into the transition intensities from state *r*→*s* (instantaneous rate of transitioning from state *r* to *s*, where *r* and *s* are adjacent states), *r* ≠ *s* and between times *t*_*ij*_ and *t*_*ij* + 1_ by using a proportional hazards framework

$$\lambda_{rsij}=\lambda_{rs}\exp\left(\beta_{rs}^{\prime }z_{ij}\right)$$

where *λ*_*rs*_ is a constant baseline intensity for the *r*→*s* transitions and ***β***_*rs*_ is a vector of regression coefficients for the associated covariates.

When all states are observable, the likelihood for time‐homogeneous Markov processes is easily computed 12. In our context, this would be the case if we knew when observations of no disability correspond to *D*_0_ or *R*, that is, if *X*(*t*_*ij*_) = *R* and *X*(*t*_*ij*_) = *D*_0_ are observed. For this situation, the likelihood contribution from patient *i*, *L*_*i*_(***Θ***) (***Θ*** is a vector of unknown parameters), conditional on their initial state *X*_*i*_(*t*_*i*0_) = *s*_*i*0_ (conditioning on the initial state is usual when it is not of interest) is

(1) $$\begin{array}{cc} L_{i}(\Theta ) & =P\left(X(t_{i1})=s_{i1},\ldots ,X(t_{im_{i}})=s_{im_{i}}|X(t_{i0})=s_{i0};\lambda_{i1},\ldots ,\lambda_{i_{m_{i}-1}}\right)\\ =\prod_{j=0}^{m_{i}-1}P(X(t_{ij+1})=s_{ij+1}|X(t_{ij})=s_{ij};\lambda_{ij})\\ =\prod_{j=0}^{m_{i}-1}P(X(t_{ij+1}-t_{ij})=s_{ij+1}|X(0)=s_{ij};\lambda_{ij})\\ :=\prod_{j=0}^{m_{i}-1}p_{s_{ij}s_{ij+1}}(t_{ij+1}-t_{ij};\lambda_{ij}) \end{array}$$

where the second and third lines follow from the Markov and time‐homogeneity property, respectively. Here, ***λ***_*ij*_ = *V* *e* *c*({*λ*_*rsij*_}_*rs*_), where *r* and *s* are adjacent states and *V* *e* *c* is a function mapping a subset of elements to a vector, and $p_{s_{ij}s_{ij+1}}(t_{ij+1}-t_{ij};\lambda_{ij}):=P(X(t_{ij+1}-t_{ij})=s_{ij+1}|X(0)=s_{ij};\lambda_{ij})$ represents the probability of transitioning from state *s*_*ij*_ to *s*_*ij* + 1_ in time *t*_*ij* + 1_ − *t*_*ij*_. For continuous‐time Markov processes, it is well known that $p_{s_{ij}s_{ij+1}}(t_{ij+1}-t_{ij};\lambda_{ij})$ can be computed as the (*s*_*ij*_,*s*_*ij* + 1_)th entry of

$$\exp(Q_{ij}(t_{ij+1}-t_{ij}))$$

where

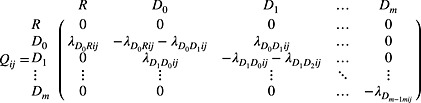

and exp() represents the matrix exponential.

In practice, observations of no disability are never known to correspond to resolved (and not immediately known to correspond to temporary non‐disability), that is, *X*(*t*_*ij*_) = *R* can never be observed. Moreover, we can only observe $X^{o}(t_{ij})=0:=\{X(t_{ij})=R\}\cup \{X(t_{ij})=D_{0}\}$, that is, overall no disability, or *X* ^*o*^(*t*_*ij*_): = *X*(*t*_*ij*_) = *D*_*k*_ when *k* > 0, as before. Here, *X* ^*o*^(*t*) denotes the observable disability process at time *t*. In order to fit the model with state space displayed in Figure 3 under this observation scheme, we construct the likelihood contribution from patient *i*, that is, *L*_*i*_(***Θ***), conditional on their initial state $X^{o}(t_{i0})=s_{i0}^{o}$, $s_{ij}^{o}\in \{0,D_{1},\ldots ,D_{m}\}$, as

(2) $$\begin{array}{cc} L_{i}(\Theta )= & \;P\left(X^{o}(t_{i1})=s_{i1}^{o},\ldots ,X^{o}(t_{im_{i}})=s_{im_{i}}^{o}|X^{o}(t_{i0})=s_{i0}^{o};\lambda_{i1},\ldots ,\lambda_{i_{m_{i}-1}}\right)\\ = & \sum_{x_{im_{i}}\in \Omega_{im_{i}}}\ldots \sum_{x_{i1}\in \Omega_{i1}}P\left(X(t_{i1})=x_{i1},\ldots ,X(t_{im_{i}})=x_{im_{i}}|X^{o}(t_{i0})=s_{i0}^{o};\lambda_{i1},\ldots ,\lambda_{i_{m_{i}-1}}\right)\\ = & \sum_{x_{im_{i}}\in \Omega_{im_{i}}}\ldots \sum_{x_{i1}\in \Omega_{i1}}\sum_{x_{i0}\in \Omega_{i0}}P\left(X(t_{i1})=x_{i1},\ldots ,X(t_{im_{i}})=x_{im_{i}}|X(t_{i0})=x_{i0};\lambda_{i1},\ldots ,\lambda_{i_{m_{i}-1}}\right)\\ \times P\left(X(t_{i0})=x_{i0}|X^{o}(t_{i0})=s_{i0}^{o}\right)\\ = & \sum_{x_{im_{i}}\in \Omega_{im_{i}}}\ldots \sum_{x_{i1}\in \Omega_{i1}}\sum_{x_{i0}\in \Omega_{i0}}\prod_{j=0}^{m_{i}-1}p_{x_{ij}x_{ij+1}}(t_{ij+1}-t_{ij};\lambda_{ij})P\left(X(t_{i0})=x_{i0}|X^{o}(t_{i0})=s_{i0}^{o}\right) \end{array}$$

where

$$\Omega_{ij}=\left\{\begin{array}{cc} \left\{D_{k}\right\} & :s_{ij}^{o}=D_{k}\;\text{and}\;k>0\\ \left\{R,D_{0}\right\} & :s_{ij}^{o}=0. \end{array}\right.$$

The second line follows because no disability is a disjoint union of temporary and permanent no disability, that is, $P(\{\{X(t)=R\}\cup \{X(t)=D_{0}\}\},C)=P(X(t)=R,C)+P(X(t)=D_{0},C)$, where *C* is any event in the probability space. The third line then follows from the theorem of total probability, and the last line follows again from the Markov and time‐homogeneity property. Note that although all observations of no disability can be either resolved or temporary non‐disability, the likelihood distinguishes between these states through the transition probabilities, which encapsulates the model structure; particularly, *R* is an absorbing state and *D*_0_ is a transient state. For example, the transition probabilities will provide greater weighting to paths that contain a transition to the resolved state after the last observation of disability if many consecutive observations of zero are seen over a long time period up to the end of follow‐up. Conversely, paths that contain transitions from the resolved state will be dismissed because these paths have zero probability of occurring. Also note that for *k* > 0, $P(X(t_{i0})=x_{i0}|\{X(t_{i0})=R\}\cup \{X(t_{i0})=D_{0}\})=0$ if *x*_*i*0_ = *D*_*k*_ and $P(X(t_{i0})=x_{i0}|X(t_{i0})=D_{k})=1$ when *x*_*i*0_ = *D*_*k*_ and 0 otherwise. Thus, only $P(X(t_{i0})=x_{i0}|\{X(t_{i0})=R\}\cup \{X(t_{i0})=D_{0}\})$ when *x*_*i*0_ = *R* or *D*_0_ is unknown. This probability is introduced to avoid having to compute the transition probabilities when the initial state is only initially known to be no disability and not *R* or *D*_0_. In the illustrative example, patients are referred to the clinic because of their level of disease activity or to acquire specialised tertiary care at the clinic. It is therefore unlikely that a patient who enters the clinic with no disability has resolved; hence, for simplicity, these patients are regarded as having temporary non‐disability at their initial visit, that is, $P(X(t_{i0})=D_{0}|\{X(t_{i0})=R\}\cup \{X(t_{i0})=D_{0}\})=1$. Note that this assumption is not relevant for patients who acquire disability at a later point in time because these patients (by definition) must have had temporary non‐disability at their initial clinic visit.

The model is fitted by maximising the complete data likelihood, which is obtained by taking the product of all likelihood contributions from each patient. Conveniently, this can be performed in the R 13 package msm 14 (see the Appendix for details), and this is one of the advantages of the proposed methodology. Along with maximum likelihood estimates, the msm package also provides 95*%* Wald intervals (which are exponentiated for baseline intensities as log baseline intensities are estimated), which will be reported together with parameter estimates (unless stated otherwise) throughout this paper.

## 3 Simulation study

This section utilises a small‐scale simulation study to help illustrate the expected discrepancies that result from knowing and not knowing if resolution has occurred. If the same model structure is specified, we would expect similar parameter estimates even if it cannot be known if resolution has occurred. Mathematically, this can be seen by comparing the likelihood in both of these situations (Equations ((1)) and ((2))); they are identical before the last observations of disability. However, because no definite information on transitions from state *D*_0_ to state *R* (or remaining in state *D*_0_) is being provided after the last observations of disability if resolution is not known, we would expect greater uncertainty about the parameters associated with the *D*_0_→*R* and *D*_0_→*D*_1_ transition intensities. Furthermore, if resolution is not known, we would expect the uncertainty with regard to the parameters governing the *D*_0_→*R* transition intensity to be heavily dependent on the lengths of follow‐up after the last observations of disability. This is because transitions from *D*_0_→*R*, although not known if they occur, can only transpire after the last observations of disability, therefore shorter time periods after these points will result in greater uncertainty in distinguishing between remaining in a state of temporary non‐disability and transitioning to resolved. In contrast, information on *D*_0_→*D*_1_ transitions contribute unequivocally to the likelihood; when transitions from no disability to disability occur. The uncertainty about the parameters governing these transitions will therefore be less affected by the follow‐up after the last observations of disability.

**Figure 4:**
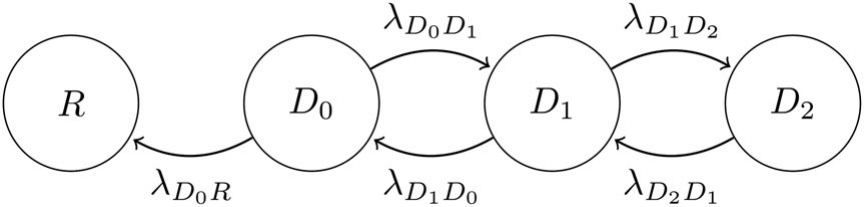
multistate diagram with transient states of disability and an absorbing resolved state.

Consider the multistate process in Figure 4, where *D*_0_ and *R* represent states of temporary and permanent non‐disability, respectively, and as an example, *D*_1_ and *D*_2_ represent states of mild/moderate and severe disability, respectively. From this process, longitudinal data from 480 patients were generated with each of the transient states containing an equal number of patients (i.e. 160) at the initial clinic visit. The simulations were based on annual clinic visits for each patient with an 8‐year follow‐up period. Furthermore, with the exception of the transition intensity from *D*_0_→*R*, the transition intensities were assumed to be modulated by a binary variable *z* = {0,1}, which has regression coefficient of *β* = 1 for each transition intensity. No covariates were assumed to act on the *D*_0_→*R* transition intensity so that its identifiability could be more simply investigated. The distribution of this binary variable was supposed to be equal between the populations at each initial state. Finally, two baseline transition intensity matrices (the (*r*,*s*)th entry corresponds to *λ*_*rs*_) were considered:

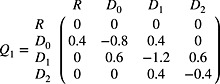

and

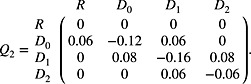

These correspond to two different scenarios. In the first scenario, the baseline intensities were all relatively large in magnitude. We would therefore expect a reasonable proportion of patients to resolve towards the start of their follow‐up. In the second scenario, the baseline intensities, particularly those from the temporary non‐disability state, were much smaller in magnitude. We would therefore expect most patients to either remain in the transient states or resolve towards the end of their follow‐up. In the Appendix, we show that the expected proportion of patients who have resolved by the end of the 8‐year follow‐up period is 0.71 and 0.22 in Scenarios 1 and 2, respectively.

In both scenarios (represented by *Q*_1_ and *Q*_2_), 500 data sets were generated under the multistate process of Figure 4. For each generated data set, the multistate process of Figure 4 was fitted under the assumption that (i) the type of non‐disability, that is, temporary non‐disability or resolved, was known; simulated values of *R* and *D*_0_ were left unaltered, and the model was fitted using Equation ((1)); and (ii) unknown; simulated values of *R* and *D*_0_ were replaced by a 0 score and fitted using Equation ((2)). These situations will be denoted as complete observation and partial observation, respectively. The mean of the parameter estimates and the estimated 95*%* Wald intervals are reported.

From Table 1, it can be seen that under Scenario 1, the mean parameter estimates of the two models are very similar to one another. The mean estimated confidence intervals of the parameters are also comparable except in the cases of the *D*_0_→*R* and *D*_0_→*D*_1_ transitions where the estimated confidence intervals are wider in the partial observation case. Nevertheless the estimate ${\hat{\lambda }}_{D_{0}R}$ is identifiable. In general, where there is sufficient amounts of follow‐up after the last observations of disability, it would be expected for reasons previously discussed that similar characteristics to that of Scenario 1 would result. In Scenario 2, it can also be seen from Table 2 that similar parameter estimates result. However, because of the short relative follow‐up after the last observations of disability, the estimate ${\hat{\lambda }}_{D_{0}R}$ in the partial observation case has a considerable amount of uncertainty associated with it; the mean estimated confidence interval of (0.026, 25) associated with this intensity is extremely wide. This has essentially resulted from the information content of the likelihood being insufficiently informative to distinguish between remaining in state *D*_0_ and transitioning to state *R* with little uncertainty. Generally, for similar situations (short follow‐up after the last disability observations), such results would also be expected. It is therefore imperative to verify empirically that a data set contains the sufficient follow‐up information if the transition intensity to the resolved state is to be identified.

**Table 1:** Mean parameter and 95% Wald interval estimates under Scenario 1.

Parameters	True	Complete observation	Partial observation
$\lambda_{D_{0}R}$	0.4	0.4 (0.36, 0.45)	0.4 (0.34, 0.48)
$\lambda_{D_{0}D_{1}}$	0.4	0.4 (0.31, 0.51)	0.4 (0.29, 0.54)
$\lambda_{D_{1}D_{0}}$	0.6	0.6 (0.5, 0.72)	0.6 (0.5, 0.72)
$\lambda_{D_{1}D_{2}}$	0.6	0.61 (0.5, 0.74)	0.61 (0.5, 0.74)
$\lambda_{D_{2}D_{1}}$	0.4	0.41 (0.34, 0.48)	0.41 (0.34, 0.48)
$\beta_{D_{0}D_{1}}$	1	1.01 (0.65, 1.37)	1.02 (0.59, 1.45)
$\beta_{D_{1}D_{0}}$	1	1.01 (0.72, 1.3)	1.02 (0.71, 1.33)
$\beta_{D_{1}D_{2}}$	1	1 (0.67, 1.34)	1 (0.67, 1.34)
$\beta_{D_{2}D_{1}}$	1	1 (0.71, 1.3)	1 (0.71, 1.3)

**Table 2:** Mean parameter and 95% Wald interval estimates under Scenario 2.

Parameters	True	Complete observation	Partial observation
$\lambda_{D_{0}R}$	0.06	0.06 (0.047, 0.076)	0.062 (0.026, 25)
$\lambda_{D_{0}D_{1}}$	0.06	0.061 (0.043, 0.087)	0.062 (0.04, 0.094)
$\lambda_{D_{1}D_{0}}$	0.08	0.081 (0.06, 0.11)	0.081 (0.06, 0.11)
$\lambda_{D_{1}D_{2}}$	0.08	0.08 (0.059, 0.11)	0.08 (0.059, 0.11)
$\lambda_{D_{2}D_{1}}$	0.06	0.059 (0.043, 0.082)	0.059 (0.043, 0.082)
$\beta_{D_{0}D_{1}}$	1	1 (0.58, 1.43)	1.01 (0.57, 1.45)
$\beta_{D_{1}D_{0}}$	1	1 (0.63, 1.36)	1 (0.63, 1.36)
$\beta_{D_{1}D_{2}}$	1	1.02 (0.65, 1.39)	1.02 (0.647, 1.39)
$\beta_{D_{2}D_{1}}$	1	1.03 (0.64, 1.42)	1.03 (0.638, 1.42)

## 4 Application

### 4.1 Estimating transition intensities

In an earlier study that describes the longitudinal course of disability, Husted *et al.* 15 considered a three‐state model to represent transitions between the categories [0,0.5), [0.5,1.5] and (1.5,3] of HAQ scores. These states corresponded to mild, moderate and severe disability, and this analysis was performed on 341 PsA patients. Since this research was conducted, the number of patients, and crucially, the follow‐up of the earlier patients have greatly increased. Therefore, an analysis that includes the possibility of a resolved state is now more meaningful. In accordance with the previous study, a multistate model with categorisations *R* (resolved state, HAQ = 0), *D*_0_ (temporary non‐disability, HAQ = 0), *D*_1_ = (0,0.5), *D*_2_ = [0.5,1.5] and *D*_3_ = (1.5,3] is now considered, and the developed methodology from the previous sections is adopted.

At each clinic visit, as well as the HAQ score, various other information was collected and so can be included in the analysis as covariates. The mean arthritis duration and age at arthritis onset during the first HAQ assessment of the 597 PsA patients were 9 years and 2 months (SD of 8 years and 8 months) and 36 years and 7 months (SD of 12 years and 11 months). The mean and median number of clinically damaged joints were 5 and 0, respectively, with an IQR from 0 to 5. Furthermore, 41*%* of the patients were women.

At baseline, 132 (22.1*%*), 243 (40.7*%*) and 90 (15.1*%*) patients were observed to be in the states *D*_1_, *D*_2_ and *D*_3_, respectively. The remaining 132 (22.1*%*) patients were observed to have no disability at baseline, of which 44 patients were never observed with disability throughout their follow‐up. Our assumption that patients can only resolve whilst in the clinic will therefore only be relevant for these 44 patients. Table 3 presents the results of fitting the five‐state model to these data. From the table, it can be seen that there is some evidence of strong influential covariate effects on the various transition intensities. Particularly, males in the mild disability state seem to have a faster transition intensity to temporary non‐disability than females. A greater number of damaged joints decreases the transition intensity from severe to moderate disability. Being diagnosed with arthritis at an older age decreases the transition intensity from mild disability to temporary non‐disability. Finally, longer duration of arthritis decreases the transition intensities between moderate and severe disability and from mild to temporary non‐disability but increases the transition intensity from moderate to severe disability. The transition intensity from state *D*_0_→*R* is estimated as 0.039 with a confidence interval of (0.024,0.065), which suggest that the model was able to identify this parameter. The likelihood ratio test statistic for the hypothesis $\lambda_{D_{0}R}=0$ was then calculated. This was compared with a 50:50 mixture of a $\chi_{1}^{2}$ distribution and a point mass at zero as this is a test of a parameter being on the boundary of its parameter space. The resulting *p*‐value was <0.001, hence inferring that a subpopulation of patients from this data have likely transitioned into a resolved state.

**Table 3:** Parameter estimates related to associations with transition intensities between disability and resolved states in 597 psoriatic arthritis patients. Also displayed are the 95% Wald intervals.

		Five‐state model		
Baseline intensities	Males versus females	Damaged joints	Age at arthritis onset (years)	Arthritis duration (years)
$\lambda_{D_{0}R}$ = 0.039 (0.024, 0.065)				
$\lambda_{D_{0}D_{1}}$ = 0.49 (0.24, 1)	− 0.16 ( − 0.54, 0.22)	0.018 ( − 0.0017, 0.038)	0.0027 ( − 0.014, 0.02)	− 0.0049 ( − 0.027, 0.017)
$\lambda_{D_{1}D_{0}}$ = 1.03 (0.53, 1.98)	0.75 (0.41, 1.09)	0.0029 ( − 0.014, 0.02)	− 0.016 ( − 0.031, − 0.0011)	− 0.032 ( − 0.053, − 0.012)
$\lambda_{D_{1}D_{2}}$ = 0.92 (0.49, 1.73)	− 0.36 ( − 0.67, − 0.048)	0.0047 ( − 0.0081, 0.018)	0.0009 ( − 0.013, 0.015)	− 0.037 ( − 0.056, − 0.017)
$\lambda_{D_{2}D_{1}}$ = 0.75 (0.41, 1.4)	0.066 ( − 0.22, 0.36)	− 0.014 ( − 0.026, ‐0.0017)	− 0.0077 ( − 0.02, 0.0047)	− 0.033 ( − 0.05, − 0.016)
$\lambda_{D_{2}D_{3}}$ = 0.074 (0.029, 0.19)	− 0.17 ( − 0.6, 0.26)	− 0.0088 ( − 0.024, 0.0063)	0.0026 ( − 0.016, 0.022)	0.029 (0.0082, 0.051)
$\lambda_{D_{3}D_{2}}$ = 0.63 (0.29, 1.39)	0.28 ( − 0.11, 0.67)	− 0.017 ( − 0.03, − 0.0041)	− 0.014 ( − 0.03, 0.00084)	0.0067 ( − 0.014, 0.027)
− 2 × Log‐likelihood = 5781.93				

### 4.2 Estimating probabilities of being resolved

Another quantity of clinical interest is the estimated probability that a given patient with a given initial state, *s*_0_, has resolved before *t* years, that is, ${\hat{p}}_{s_{0}R}(t)$. From the fitted model in Section 4.1, it is difficult to obtain valid estimates of $p_{s_{0}R}(t)$, particularly if in the interval [0,*t*], it is challenging to represent reasonably the evolution of the time‐varying covariate number of damaged joints and to a lesser extent arthritis duration. Arthritis duration is less problematic because it can be updated with time because of its deterministic nature; however, the number of damaged joints is a random stochastic process and therefore would require joint modelling with the disability process 16 if $p_{s_{0}R}(t)$ is to be reasonably estimated. For illustrative purposes, we fit another joint model with outcomes as before (described by states {*R*,*D*_0_,*D*_1_,*D*_2_,*D*_3_}) but with covariates sex, age at onset of arthritis (years) and arthritis duration categorised as follows: less than 2 years, between 2 and 5 years inclusive and greater than 5 years, thus allowing arthritis duration to be more easily updated (see the Appendix Table A.1 for the results). This model is then less problematic for estimating $p_{s_{0}R}(t)$.

Consider at clinic entry a male patient who was diagnosed with arthritis at 36 years and 7 months and has arthritis duration of 9 years and 2 months (covariates set to be their means). Table 4 displays the results of the estimated probabilities of resolution and their respective 95*%* confidence intervals (calculated using the technique described in 17), conditional on various initial states, before *t* = 5, 10 and 15 years. Table 4 demonstrates that this illustrative patient, if he had entered the clinic with temporary non‐disability, has an estimated probability of 0.11 (0.067, 0.17), 0.18 (0.11, 0.26) and 0.23 (0.14, 0.35) of being resolved before 5, 10 and 15 years, respectively. However, these probabilities decrease to 0.013 (0.007, 0.022), 0.058 (0.035, 0.091) and 0.11 (0.067, 0.18) if he had been diagnosed with severe disability upon clinic entry.

**Table 4:** Estimated probabilities and corresponding 95% confidence intervals of resolving before t = 5, 10 and 15 years for a male patient who was diagnosed with arthritis at 36 years and 7 months and with arthritis duration of 9 years and 2 months.

Initial state (years)	*D*_0_	*D*_1_	*D*_2_	*D*_3_
5	0.11 (0.067, 0.17)	0.07 (0.042, 0.12)	0.029 (0.017, 0.049)	0.013 (0.007, 0.022)
10	0.18 (0.11, 0.26)	0.14 (0.085, 0.21)	0.086 (0.054, 0.13)	0.058 (0.035, 0.091)
15	0.23 (0.14, 0.35)	0 .19 (0.12, 0.3)	0.14 (0.086, 0.22)	0.11 (0.067, 0.18)

## 5 Discussion

Intermittently observed multistate processes that are subject to resolution commonly occur in many areas of health research. In these setting, we propose a joint modelling approach whereby both the multistate and resolution processes are combined via an expanded multistate model. In this framework, the intermittent observations are naturally handled, and by considering observations of no disability to be in either the resolved state or temporary non‐disability state, the uncertainty arising from not knowing if and when the process of interest has resolved is captured. Conceptually, this involves considering all possible paths, which may or may not enter the resolved state at any instance that give rise to the intermittent observations. Such an analysis is often difficult to undertake; however, because of the readily available multistate modelling software, this approach is particularly appealing. One use of this joint model would be to provide a means for considering the extent to which a subpopulation of patients have resolved. If such a population is seen as likely, it is straightforward to further incorporate covariates in the transition intensity to the resolved state.

The results of Section 3 indicate clearly that care must be exercised in the use of this model. In particular, the data on which the model is based must suggest that it is empirically plausible for a subpopulation to have resolved. Failure of this possibility will result in identification problems even if a subpopulation of patients have resolved. Another concern is the clinical plausibility of a resolved population. The use of this model for disease processes where resolution is unrealistic would be very difficult to defend.

Although understanding the disability process was the primary aim of Section 4.1, thus important time‐varying (uncategorised) covariates were incorporated into the analysis, Section 4.2 highlights the difficulty of such an analysis if estimating the probability of resolving before a specified time, *t*, is of interest. In particular, in the interval [0,*t*], it can be challenging to accurately represent the evolution of time‐varying covariates. Section 4.2 therefore utilised another joint model without the covariate number of damaged joints and with arthritis duration categorised to illustrate how the relevant probabilities can be estimated reasonably. An interesting area of future research would be to consider the possibility of directly modelling the transition probabilities 18, specifically the probability of resolving before a specified time point, instead of the transition intensities. In addition to estimating transition probabilities for specific patients, this would also provide more interpretable covariate effects on these probabilities.

Finally, the estimated transition intensity to a resolved state will depend on the model specification for the disability process. In the illustrative example, strong modelling assumptions (Markov time‐homogeneous) were made. Future work that relaxes these assumptions would be useful.

## Appendix B

### B.1

In this section, we compute the expected proportion of patients who are expected to have resolved by the end of the 8‐year follow‐up period. As there are 160 patients in each initial state (except from state *R*), with 80 patients such that *z* = 1 and 80 patients such that *z* = 0, the expected proportion of patients in state *R* at the end of the 8‐year follow‐up period is

$$\underset{k\in \{D_{0},D_{1},D_{2}\}}{\sum }p_{kR}(8|z=1)/6+\underset{k\in \{D_{0},D_{1},D_{2}\}}{\sum }p_{kR}(8|z=0)/6.$$

In Scenarios 1 and 2, this quantity is calculated as 0.71 and 0.22, respectively.

**Table A.1:** Parameter estimates related to associations with transition intensities between disability and resolved states in 597 psoriatic arthritis patients. Also displayed are the 95% Wald intervals.

		Five‐state model		
Baseline intensities	Males versus females	Age at arthritis onset (years)	Arthritis duration [2,5] vs < 2	Arthritis duration > 5 vs < 2
$\lambda_{D_{0}R}$ = 0.039 (0.023, 0.065)				
$\lambda_{D_{0}D_{1}}$ = 0.57 (0.22, 1.53)	− 0.15 ( − 0.52, 0.23)	0.0041 ( − 0.012, 0.02)	− 0.74 ( − 1.63, 0.16)	− 0.14 ( − 0.91, 0.63)
$\lambda_{D_{1}D_{0}}$ = 1.11 (0.48, 2.54)	0.82 (0.48, 1.17)	− 0.014 ( − 0.029, ‐0.00024)	− 0.55 ( − 1.31, 0.2)	− 0.67 ( − 1.29, − 0.051)
$\lambda_{D_{1}D_{2}}$ = 1.1 (0.45, 2.55)	− 0.36 ( − 0.67, − 0.06)	0.0067 ( − 0.0063, 0.02)	− 0.26 ( − 1.15, 0.62)	− 1 ( − 1.72, − 0.28)
$\lambda_{D_{2}D_{1}}$ = 0.91 (0.42, 2)	0.025 ( − 0.25, 0.3)	− 0.00093 ( − 0.013, 0.011)	− 0.8 ( − 1.55, − 0.043)	− 1.16 ( − 1.76, − 0.57)
$\lambda_{D_{2}D_{3}}$ = 0.29 (0.081, 1.02)	− 0.21 ( − 0.63, 0.2)	− 0.012 ( − 0.03, 0.0061)	0.24 ( − 0.85, 1.33)	− 0.53 ( − 1.51, 0.46)
$\lambda_{D_{3}D_{2}}$ = 2.3 (0.77, 6.9)	0.34 ( − 0.05, 0.73)	− 0.022 ( − 0.038, − 0.0066)	− 0.48 ( − 1.37, 0.42)	− 1.31 ( − 2.11, − 0.51)
− 2 × Log‐likelihood = 5814.76				
